# Simple and Quick Visualization of Periodical Data Using Microsoft Excel

**DOI:** 10.3390/mps2040081

**Published:** 2019-10-11

**Authors:** Hideaki Oike, Yukino Ogawa, Katsutaka Oishi

**Affiliations:** 1 Food Research Institute, National Agriculture and Food Research Organization (NARO), Tsukuba, Ibaraki 305-8642, Japan; yukino.ogawa@affrc.go.jp; 2 Biological Clock Research Group, Biomedical Research Institute, National Institute of Advanced Industrial Science and Technology (AIST), Tsukuba, Ibaraki 305-8566, Japan; k-ooishi@aist.go.jp; 3 Department of Applied Biological Science, Graduate School of Science and Technology, Tokyo University of Science, Noda, Chiba 278-8510, Japan; 4 Department of Computational Biology and Medical Sciences, Graduate School of Frontier Sciences, The University of Tokyo, Kashiwa, Chiba 277-8562, Japan; 5 School of Integrative and Global Majors (SIGMA), University of Tsukuba, Tsukuba, Ibaraki 305-8577, Japan

**Keywords:** actogram, heatmap, biological rhythm, circadian clock, chronobiology, sleep pattern, ECG, EEG

## Abstract

Actograms are well-established methods used for visualizing periodic activity of animals in chronobiological research. They help in the understanding of the overall characteristics of rhythms and are instrumental in defining the direction of subsequent detailed analysis. Although there exists specialized software for creating actograms, new users such as students and researchers from other fields often find it inconvenient to use. In this study, we demonstrate a fast and easy method to create actograms using Microsoft Excel. As operations in Excel are simple and user-friendly, it takes only a few minutes to create an actogram. Using this method, it is possible to obtain a visual understanding of the characteristics of rhythms not only from typical activity data, but also from any kind of time-series data such as body temperature, blood sugar level, gene expressions, sleep electroencephalogram, heartbeat, and so on. The actogram thus created can also be converted to the "heatogram” shown by color temperature. As opposed to conventional chronograms, this new type of chronogram facilitates easy understanding of rhythmic features in a more intuitive manner. This method is therefore convenient and beneficial for a broad range of researchers including students as it aids in the better understanding of periodic phenomena from a large amount of time-series data.

## 1. Introduction

There are many rhythmic events in living organisms [1]. A typical example is the circadian rhythm, which is the approximately 24-hour activity rhythm found in numerous animals and plants as well as some bacteria. In addition, there are some shorter rhythms, such as ultradian rhythm, which consists of a few hours of oscillation, activities repeated in seconds such as heartbeat, and activities repeated in milliseconds such as nerve firing. In the field of chronobiological research, activity patterns such as circadian rhythms and circatidal rhythms are often represented using actograms. Actograms are suitable for visualizing periodic activities, thereby enabling easy understanding of transition of period length and the characteristics [2]. However, the use of actograms, as the name suggests, is limited to activity rhythms, and despite its usefulness, researchers other than chronobiologists rarely use it.

Usually, actograms are created using professional software, which is often packaged with the behavior analysis system of animals, such as ClockLab (Actimetrics) and Chronobiology kit (Stanford Software Systems) or free software provided by chronobiologists [3,4,5]. While these various software packages provide advanced analysis tools, they are often inconvenient for new users such as students and researchers in other research fields. For example: 1) the packaged software with an analytical device is expensive, especially for multiuser licenses. 2) The operation methods differ depending on the software, and the settings and tools are not always user-friendly. 3) It is difficult to freely customize colors, shading, axis labels, size, and so forth to fit the presentation styles. As a result, students and researchers in other fields do not get to the point of understanding what the actogram means. In contrast, many researchers including students routinely use Excel for their data analysis. If the data can be easily represented in a visual manner with the function of Excel, it is easy to understand and operate the actograms. Here, we demonstrate quick and easy methods to draw classical actograms using Excel, as well as introduce a novel type of chronogram (heatogram) that is created using color temperature. If further analysis of rhythmic characteristics is required, free software solutions such as Actogram J [3] and RhythmicAlly [5] may be useful.

## 2. Experimental Design

In chronobiological research, which involves 24-hour monitoring of biomarkers in the medical field, acquisition of data over time is crucial. For example, when evaluating the circadian rhythm of behavior in mice, continuous data acquired by an infrared sensor or a rotating wheel is often used [6]. Usually, several minutes of activity data are recorded over several weeks, resulting in more than tens of thousands of points of data per mouse. When data visualization is done through actograms, understanding the rhythmic phenomena becomes easier to understand. Here, we present a method for visualizing tens of thousands of data through actograms easily using Excel. Any time-series data and any periodicity, circadian, ultradian, or even several-millisecond rhythms may be used.

First, we describe a protocol that converts the activity rhythm of a mouse acquired by an infrared sensor into a conventional actogram (3.1), and a protocol that expresses it as color temperature in the form of a heatmap (3.2). Subsequently, as an application example, a visualization of body temperature data acquired every 15 min by the abdominal-cavity-embedded temperature sensor, time series of human blood sugar level for two weeks, sleep electroencephalogram (EEG), and heartbeat data from electromyograms (EMG) is created. In addition, we demonstrate a method that can be used for understanding the cycle length simply by changing the cycle of the actogram. 

As this protocol focuses on data analysis, the data acquisition methods are referred to in previous papers. All animal experiments in this study were performed according to the guidelines for animal experiments published by the National Institute of Advanced Industrial Science and Technology (AIST), or the guidelines of the Ministry of Agriculture, Forestry and Fisheries for laboratory animal studies. The studies were reviewed and approved by the AIST Animal Care and Use Committee (approval number: 2018-054) or the Animal Care and Use Committee of the National Food Research Institute (NFRI; approval number: H30-018 and H30-043). The experimental protocol dealing with human data was approved by the ethical committees of the NFRI (approval number: 31-0008). Sample data and Excel files containing visualized results are provided in the Supplementary Materials.

### Equipment

A computer installed with Microsoft Excel software (Excel 2007 or later).

## 3. Procedure

### 3.1. Simple Method of Preparation of Conventional Double-Plot Actogram by Excel (about 10 minutes)

**Figure 1:**
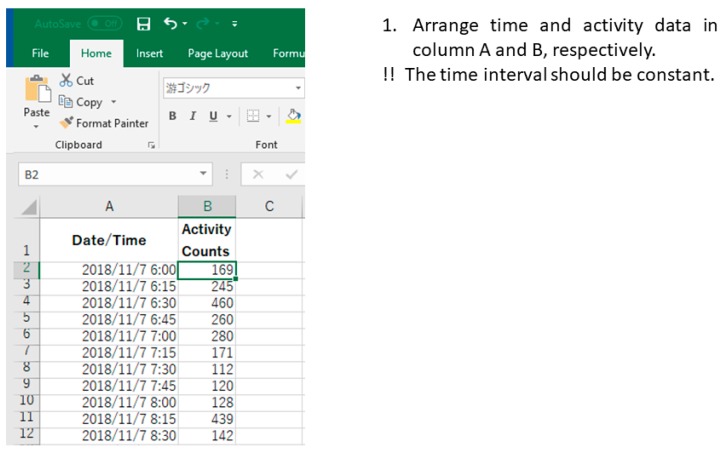
Example of data arrangement in Excel (Procedure 3.1, step 3).

1. Prepare time series of activity data (or download sample data from the Supplementary Materials).
2. Open Microsoft Excel.
3. Paste a sequence of time and activity data in column A and B, respectively (Figure 1).
4. Line up every 48 hours of data in a column.

**Figure 2:**
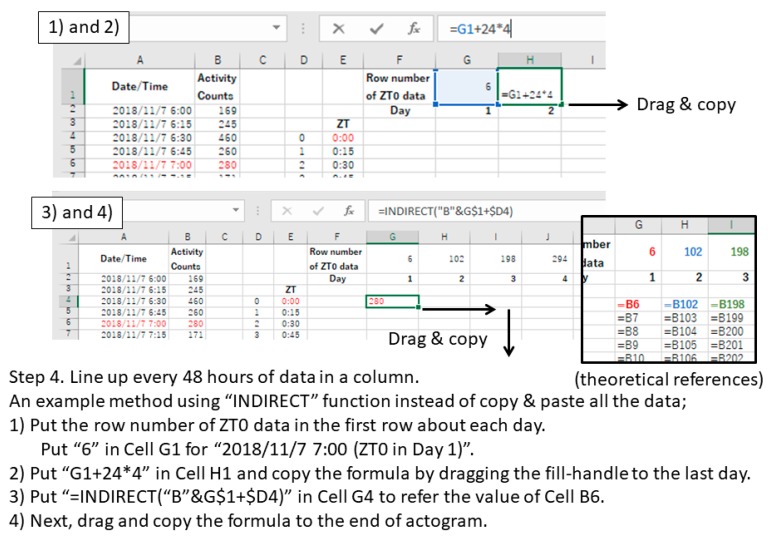
Examples of data processing in Excel (Procedure 3.1, step 4).

**Option:** “INDIRECT” or “OFFSET” function makes it easy when the amount of data is extremely large (Figure 2).

**Figure 3:**
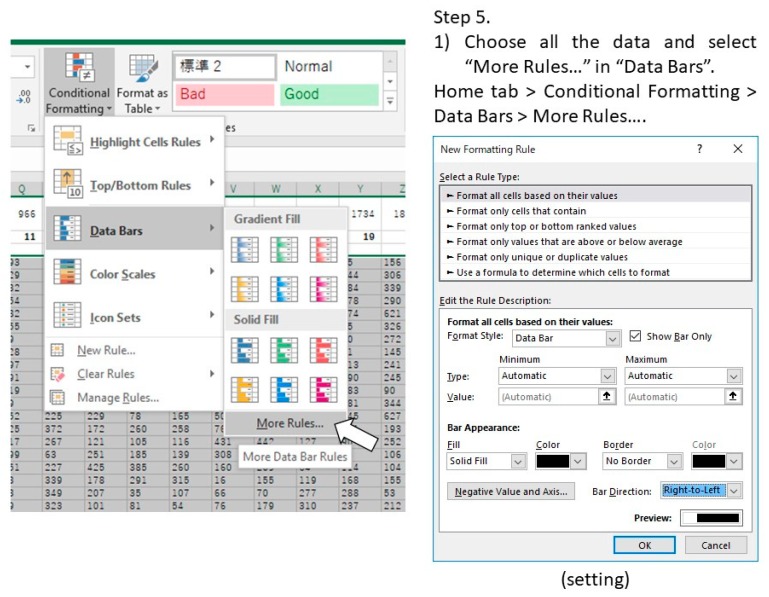
Examples of data processing in Excel (Procedure 3.1, step 5).

**Figure 4:**
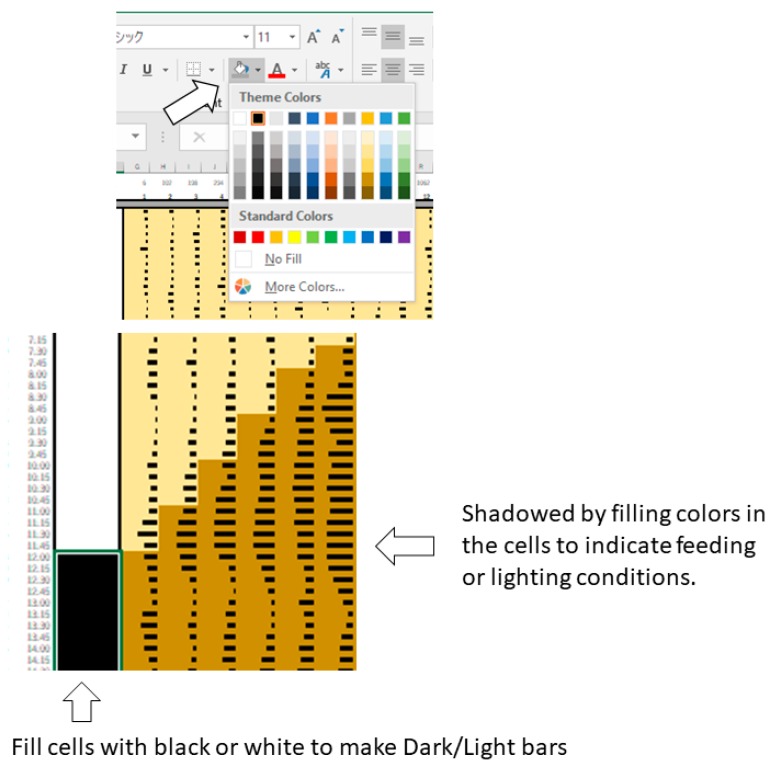
Examples of data processing in Excel (Procedure 3.1, step 6).

**Figure 5:**
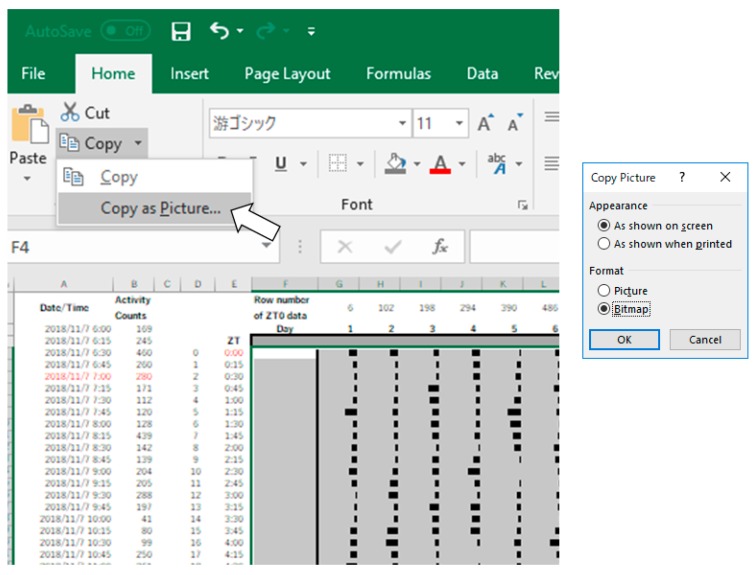
Examples of data processing in Excel (Procedure 3.1, step 7).

**Figure 6:**
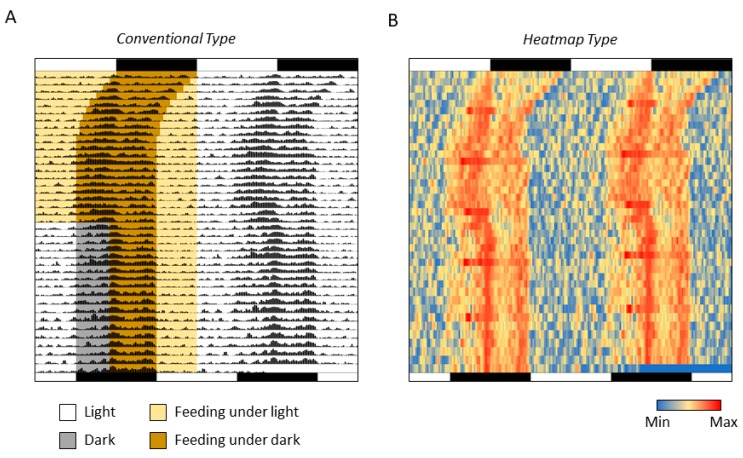
Actograms drawn by Excel. (**A**) Conventional Type; (**B**) Heatmap Type.

5. Select all the data and quantitatively visualize using “Data Bars” function (Figure 3).
6. **Optional step:** Make dark/light bars by filling the cells with black and white or make shadows by filling the cells with colors in order to indicate light/dark or feeding conditions (Figure 4).
7. Copy the actogram using “Copy as Picture” function (Figure 5).
8. Paste the actogram in a new file of image editing software such as Microsoft PowerPoint, and edit the size and orientation of the actogram (Figure 6A). The completed Excel file is provided in the Supplementary Materials as File S1.

### 3.2. Conversion to Heatmap-Type Actogram or “Heatogram” (a few minutes)

**Figure 7:**
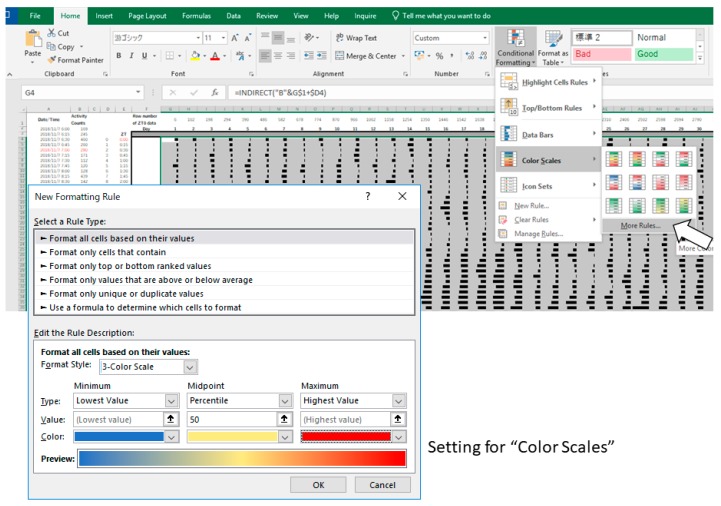
Examples of data processing in Excel (Procedure 3.2, step 5).

Amend after step 5 of the above-mentioned procedure to the following:

5. Select all the data and quantitatively visualize using “Color Scales” in the same dropdown menu (Figure 7).
6. **Optional step:** Use small gray fonts in the cells to make them less noticeable.
7. Copy the actogram using “Copy as Picture” function.
8. Paste the actogram in a new file of drawing software such as Microsoft PowerPoint, and edit size and orientation (Figure 6B). The completed Excel file is provided in the Supplementary Materials as File S1. A video file that includes the whole process of creating an actogram is also provided in the Supplementary Materials as File V1.

## 4. Expected Results

**Figure 8:**
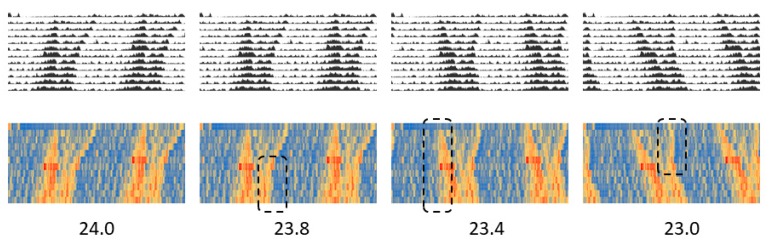
Actograms created with the period length shown at the bottom of them.

The conventional double-plot actogram drawn in Excel here is equivalent to the one drawn using other professional software. The easy addition of shadows also allows experimental conditions such as light/dark and/or feeding/fasting to be represented in the actogram concurrently, thus enabling the creation of a more easily understandable actogram. Moreover, compared to a conventional actogram, some points can be better understood when the actogram is converted into a heatogram. For example, the increase in activity once a week, which is due to cage replacement (Figure 6B), can be better observed on the heatmap-type actogram compared to a conventional one. In the actograms created using Excel, the period length can also be easily changed. By drawing several actograms with different cycle lengths, it is possible to easily understand the desired period length (Figure 8 and File S2).

**Figure 9:**
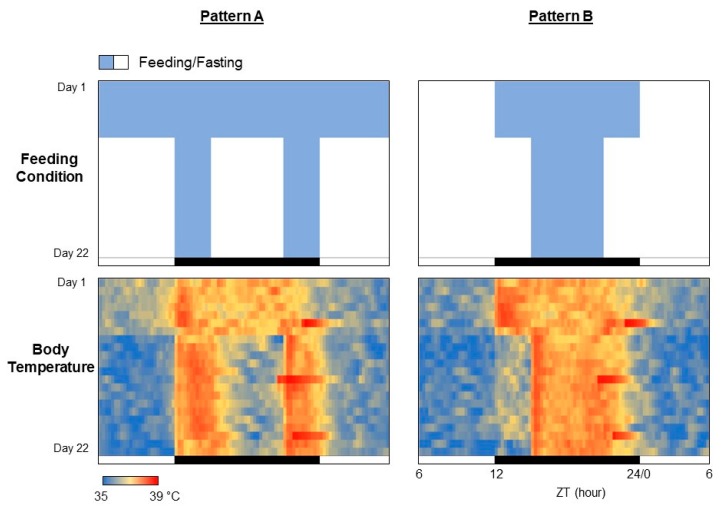
Heatmap-type chronograms based on body temperature data.

The procedure presented in this study can be easily applied to any other periodic data such as body temperature, blood sugar level, sleep pattern, blood pressure, clock gene expressions, and so on. A small temperature logger implanted into the abdominal cavity of a mouse or rat can monitor the core body temperature for several weeks [7]. Here, we demonstrate an example of the experimental result demonstrating the effects of feeding patterns on the core body temperature rhythms in a C57BL/6 mouse (Figure 9). For the feeding pattern A, the first week was free feeding, and the remaining 2 weeks limited the feeding to 3 hours from the beginning of the dark phase and 3 hours from the end of the dark phase, for a total of 6 hours (Figure 8, upper left). The feeding pattern B allowed 12 hours of feeding during the dark phase for the first week and then 6 hours of feeding for the last two weeks (Figure 8, upper right). The rhythm of core body temperature is found to be in complete agreement with the feeding patterns (Figure 8, lower panels). Incidentally, the fact that the body temperature drastically rises just before the end of the dark period on Days 6, 13, and 20 is due to the replacement of cages and food for regular maintenance.

**Figure 10:**
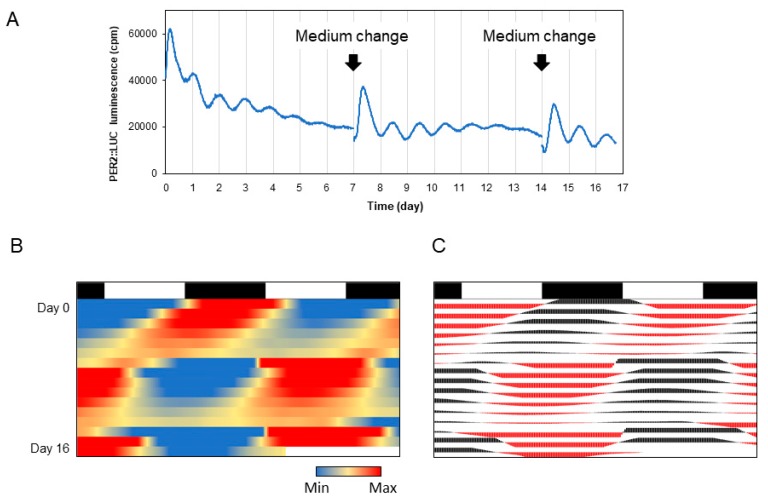
Chronograms of PER2 protein expression in cultured cells. (**A**) Time-series data of luminescence of PER2::LUC reporter in cultured neuronal cells. (**B**) Heatmap-type chronogram. (**C**) Conventional-type chronogram.

The circadian clock is also maintained under culture conditions in several cells. For example, when luciferase is fused to a clock gene and introduced into the cells, circadian rhythm can be monitored as a luminescence (Figure 10A and File S3). The protocol here is also applicable for the visualization of the periodic luminescence. There is no need for specialized software, and if continuous data of time and light values are available, they can be easily represented by creating an actogram in Excel. An example of the circadian rhythm under the culture conditions of a neuron expressing PER2::LUC gene [8] is shown in Figure 10B. 

**Figure 11:**
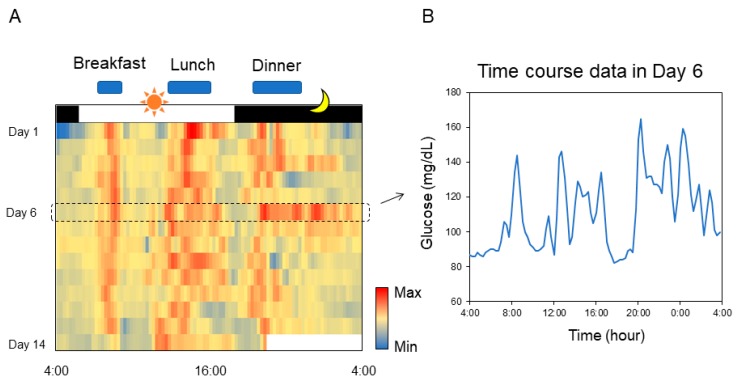
Chronogram of blood glucose. (**A**) Heatmap-type chronogram based on blood glucose level monitored by the commercially available flash glucose self-monitoring system. (**B**) Time course data of the blood glucose level on Day 6 is extracted from the chronogram.

Recently, 24-hour monitoring of blood glucose levels has become possible with commercially available sensors [9]. Daily pattern of blood glucose fluctuation is easily understood with a heatogram (Figure 11 and File S4). The time period during which the blood sugar level tends to rise or fall can also be conveniently identified. The primary advantage associated with the use of Excel is that focused data can be easily converted to a graphical representation (right graph in Figure 11). 

**Figure 12:**
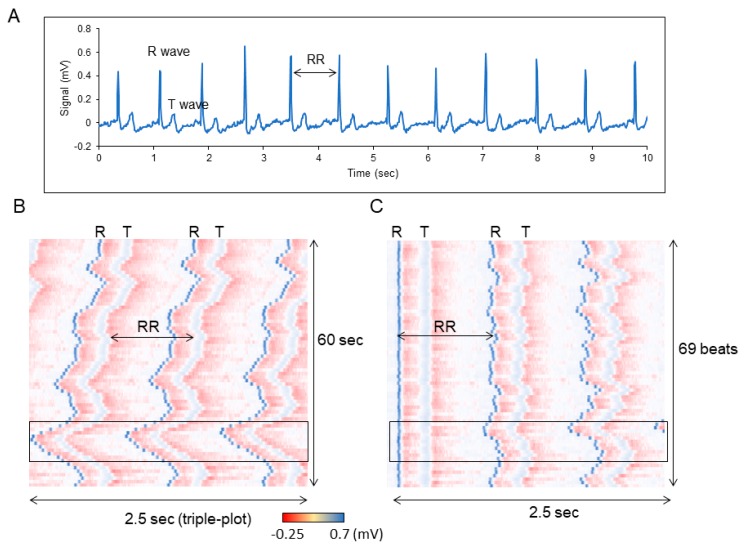
Chronogram of heartbeats. (**A**) Time course data of the heartbeats for 10 s. (**B**) Heatmap-type chronogram based on EMG data for 60 sec. (**C**) Position of each R wave is aligned.

The above-mentioned protocol can also be used to analyze shorter periodic data. For example, the beating of the heart repeats roughly once every second. Electrocardiogram (ECG) is a typical method for monitoring the heart rhythm [10]. Although, most often, the waveform data obtained from ECG is used, as shown in Figure 12A, the change in the heartbeat pattern over time is difficult to be analyzed. In addition, if the portions of each heartbeat are extracted and overlapped, the information regarding time lapse is lost. Therefore, data visualization using actogram makes it easy to observe the manner in which each rhythm changes over time (Figure 12B and File S5). In the case of a heartbeat, it can be seen that the interval between beats is easy to change. Therefore, when the peak values of R waves are aligned, the timing of the next T wave is almost the same, but it is observed that the timing to the next R wave fluctuates with time (Figure 12C). This representation makes the fluctuation of the heart rate interval easy to comprehend. As the fluctuation of the heartbeat rate interval is considered to be caused due to stress, this method can be used to visually represent various degrees of stress.

**Figure 13:**
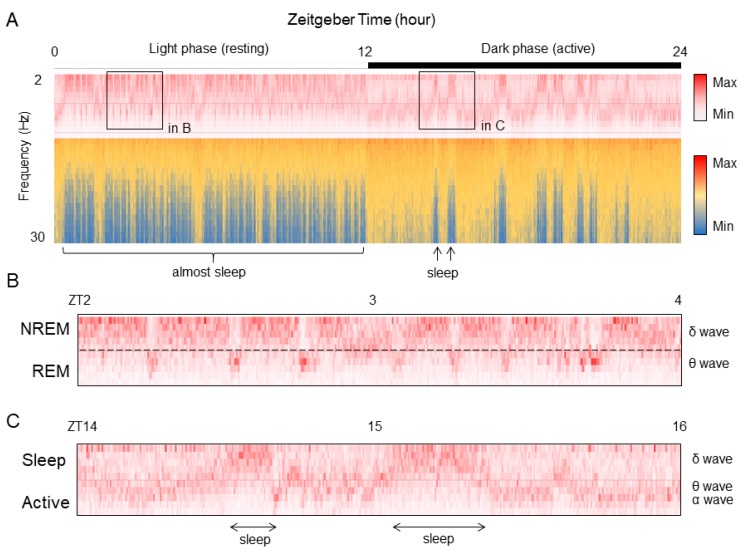
Frequency component data of EEG is visualized by Excel. (**A**) Time course data of the EEG frequency for 24 hours. (**B**) Enlarged data during sleep phase (light period) indicated area in A. (**C**) Enlarged data during active phase (dark period) indicated area in A.

Finally, an application of the method to visualize electroencephalograms (EEG) during sleep is presented. The frequency of the brain waves changes according to the state of sleep/activity [11,12]. In addition, even during sleep, delta waves are predominant in NREM (non-rapid eye movement) sleep, while the percentage of theta waves increases in REM (rapid eye movement) sleep. Sleep researchers use specialized software to combine a rate of delta waves and theta waves with electromyograms (EMG) to classify awakening, NREM sleep, and REM sleep. However, it is difficult to automate sleep classification because the threshold value is easily changed by individual. Thus, it takes time to be able to understand the sleep and wake structure from the raw data of brain waves, and information is also lost in the decision-making process. Therefore, by visualizing the difference in signal intensity according to the sleep frequency closer to the raw data with Excel as it is, it becomes possible to intuitively understand the sleep/wake pattern (Figure 13A and File S6). In this graph, the EEG data acquired at 20 seconds per epoch as the intensity value for each frequency is represented on the vertical axis, and the time duration of 24 hours is represented on the horizontal axis. As slow waves (12 Hz or less) increase and β waves (12 Hz or more) decrease during sleep, sleep and awakening patterns can be visually distinguished by focusing on the β waves. Furthermore, with regard to the difference between NREM and REM during sleep, focusing on the slow wave, the state with more slow delta waves is the NREM sleep time zone, and the time zone with higher frequency theta waves is REM sleep (Figure 13B). As the average episode length of REM sleep is about two minutes, it is observed that the time zone in which the frequency is shifted to the side of the long wavelength during NREM sleep matches with the episode of REM sleep. Additionally, in the dark phase where awakening is frequent, the frequency close to the theta wave increases, but it has a slightly higher and a longer-lasting characteristic (Figure 13C). Thus, it can be distinguished from REM sleep during the light phase.

## Supplementary Materials

The following are available online at https://www.mdpi.com/2409-9279/2/4/81/s1, File S1: Conventional- and heatmap-type actograms (Excel file). File S2: Period changeable actogram (Excel file). File S3: Chronogram of PER2::LUC luminescence (Excel file). File S4: Chronogram of blood glucose level (Excel file). File S5: Chronogram of ECG (Excel file). File S6: Chronogram of EEG (Excel file). File V1: A video file that includes whole process of creating an actogram (mp4 file).
